# First Complete Genome Sequence of *Tenacibaculum dicentrarchi*, an Emerging Bacterial Pathogen of Salmonids

**DOI:** 10.1128/genomeA.01756-15

**Published:** 2016-02-18

**Authors:** Horst Grothusen, Alejandro Castillo, Patricio Henríquez, Esteban Navas, Harry Bohle, Carolina Araya, Fernando Bustamante, Patricio Bustos, Marcos Mancilla

**Affiliations:** Laboratorio de Diagnóstico y Biotecnología, ADL Diagnostic Chile Ltda., Puerto Montt, Chile

## Abstract

*Tenacibaculum*-like bacilli have recently been isolated from diseased sea-reared Atlantic salmon in outbreaks that took place in the XI region (Región de Aysén) of Chile. Molecular typing identified the bacterium as *Tenacibaculum dicentrarchi*. Here, we report the complete genome sequence of the AY7486TD isolate recovered during those outbreaks.

## GENOME ANNOUNCEMENT

Members of the genus *Tenacibaculum* are Gram-negative, marine fish pathogens, responsible for tenacibaculosis. Ulcerative lesions on different parts of the body, especially on the skin surface as pathognomonic signs, manifest the disease (1). Long, slender, filamentous rods are the typical morphology, which are tough to isolate using standard media for marine bacteria. Standard phylogeny determined by the sequencing of the 16S rRNA gene placed several species out of the genus *Flexibacter*; therefore, the genus *Tenacibaculum* was proposed (2), which currently comprises 21 environmental and pathogenic species (3). *T. maritimum* is recognized as a major cause of tenacibaculosis in marine fish, and has been isolated from several host species worldwide (4). *Tenacibaculum* are slow growing bacteria, a trait related to the underdiagnosis of the disease based on culture techniques. Moreover, the closed phylogenetic relationship between species makes their differentiation by conventional approaches difficult. In Chile, even though there is no official report on the isolation of *T. maritimum*, the regulatory agency has informed PCR-based detections since 2012.

Outbreaks in Atlantic salmon (*Salmo salar*) reared in the XI region (Región de Aysén) occurred in the second half of 2015. Clinical findings were ascribed to tenacibaculosis, but molecular screening for *T. maritimum* was negative. *Tenacibaculum*-like bacteria were isolated as pure culture from a skin lesion. Typing by 16S rRNA sequencing revealed the presence of *T. dicentrarchi* (5). Here, we present the complete genome sequence of the *T. dicentrarchi* AY7486TD field isolate.

Sequencing was performed at Macrogen, Inc. (Seoul, South Korea) using the Pacific Bioscience single-molecule real-time (SMRT) cell 8Pac v3 and DNA polymerase binding kit P6 v2 for library preparation. 62,255 mapped reads (~13,000-bp average length) from a total of 63,904 reads were *de novo* assembled using SMRT Analysis v2.3.0.1 (http://www.pacb.com) into a circular chromosome of 2,918,253 bp (*N*_50_ = 18,803). The genome depicts a G+C content of 31.5%. The assembled data were annotated with the NCBI Prokaryotic Genome Annotation Pipeline using the best-placed reference protein set as the annotation method implemented in GeneMarkS+ revision 3 0 software. Annotated features were 2,542 genes, 2,420 coding sequences (CDSs), 22 pseudogenes, 10 rRNAs, 1 small noncoding RNA (ncRNA), and 69 tRNAs. An *oriC* region was predicted by the Ori-Finder tool (6).

A relevant feature from the annotation was the presence of genes encoding several metallopeptidases and collagen-binding proteins. These genes, along with those encoding hemolysins are likely involved in the extensive surface tissue damage observed in affected fish. Interestingly, genes encoding structural components of a type IX secretion system (T9SS) were also identified. Recent studies have shown that the T9SS not only plays a role in gliding motility, but also in the delivery of effector proteases of related bacteria (7). Further research will shed more light on these and others virulence aspects of this emerging pathogen.

This first complete genome sequence will serve to improve diagnostics and contribute to a better understanding of the biology of the *Tenacibaculum* genus.

### Nucleotide sequence accession numbers.

The sequence of the AY7486TD isolate is part of a sequencing project, which has been deposited at DDBJ/EMBL/GenBank under the accession no. CP013671. The version described in this paper is the first version, CP013671.1.
